# Assessment of the Cardiovascular Risk Profile of Infants Exposed to Pre-eclampsia *in-utero*: A Prospective Case-Control Study in South African Children of African Ancestry

**DOI:** 10.3389/fcvm.2021.773841

**Published:** 2021-11-23

**Authors:** Benedicta Ngwenchi Nkeh-Chungag, Godwill Azeh Engwa, Charles Businge, Kaltrina Kutllovci-Hasani, Andre P. Kengne, Nandu Goswami

**Affiliations:** ^1^Department of Biological and Environmental Sciences, Faculty of Natural Sciences, Walter Sisulu University PBX1, Mthatha, South Africa; ^2^Department of Obstetrics and Gynaecology, Nelson Mandela Academic Hospital, Walter Sisulu University, Mthatha, South Africa; ^3^Division of Obstetrics, Medical University of Graz, Graz, Austria; ^4^Non-communicable Diseases Research Unit, South African Medical Research Council, Cape Town, South Africa; ^5^Physiology Division, Otto Loewi Research Center for Vascular Biology, Immunology and Inflammation, Medical University of Graz, Graz, Austria; ^6^College of Medicine, Mohammed Bin Rashid University of Medicine and Health Sciences, Dubai, United Arab Emirates

**Keywords:** pre-eclampsia (PE), cardiovascular risk factor, vascular dysfunction, African ancestry, blood pressure

## Abstract

**Background:** It has been reported that maternal gestational environment may be programmed to have a significant impact on foetal and offspring health later in life. Studies have shown that children born to pre-eclamptic mothers are prone to obesity, hypertension, and diabetes in their adult life. However, such findings are yet to be established in an African population. This protocol is for a study aiming to investigate the relationship between pre-eclampsia (PE) and cardiovascular risk in children born to pre-eclamptic mothers in a South African population of African descents.

**Methods:** A prospective case-control design will be employed to recruit pre-eclamptic and normotensive pregnant women and their offspring after birth. Pregnant women will be assessed for cardiovascular risk factors including PE, obesity, haemodynamics, lipids, glycaemic indices, oxidative stress, and vascular function at 30 weeks of gestation. The cardiovascular risk profile of their offspring will be assessed at birth and 6 weeks later. The difference in cardiovascular risk profile between children born to the pre-eclamptic and normotensive mothers will be compared and the correlation between maternal and offspring cardiovascular risks will be investigated.

**Discussion:** This will be the first prospective study to assess the *in-utero* effect of cardiovascular risk in offspring born to pre-eclamptic women of African ancestry. It is expected that findings from this study will provide information on the cardiovascular effect of *in-utero* exposure to PE in a population of African ancestry. This knowledge will advise policy on the management of women with PE with a view of protecting cardiovascular health in offspring.

## Introduction

Hypertension is an independent modifiable risk factor for the development of cardiovascular diseases (CVDs) (1). Although hypertension is prevalent in adults and increasingly becoming prevalent in children (1), there has been documented reports of hypertension in neonates (2). The Neonatal Intensive Care Unit (NICU) report showed that hypertension may occur in up to 3% of neonates (3) while a large multi-centre study by the Worldwide Acute Kidney Injury Epidemiology in Neonates (AWAKEN) reported a 1.8% incidence rate of neonatal hypertension (4). Although the origin and major predisposing factors for high blood pressure (BP) in neonates is unknown, studies have shown that acute kidney injury (4), maternal hypertension and pre-eclampsia (PE) may be responsible (5). PE is a hypertensive disorder defined as the presence of hypertension (BP ≥ 140/90 mm Hg) on two occasions at least 4 h apart with proteinuria or severe hypertension (BP ≥ 160/110 mm Hg) with or without proteinuria occurring after 20 weeks of gestation in a previously normotensive woman (6). PE is thought to be a contributor to neonatal hypertension and has been associated with small for gestational age babies with increased risk for CVDs (7).

As suggested by the concept of the Developmental Origins of Health and Disease, the maternal gestational environment may have a significant impact on foetal health with offspring been programmed for hypertension, obesity, diabetes, and CVDs in adulthood (8). Foeto-placental vascular endothelial dysfunction may cause epigenetic alteration in the intrauterine environment of the foetus and may be the origin of chronic diseases later in life (9). Moreover, PE has been reported to be associated with foeto-placental vascular endothelial dysfunction (10, 11). More so, there is evidence that children born to pre-eclamptic mothers have higher intima media thickness, adverse cardiac remodelling and higher blood pressure (12, 13) suggesting that offspring of pre-eclamptic mothers are prone to CVDs. However, all these studies were done in non-African populations and therefore may not necessarily apply to people of African ancestry.

In South Africa, PE affects about 5% of pregnant women and 12% of primigravidae (14, 15). Although the cause(s) of PE remain unclear, it is thought to stem from placental malfunction during pregnancy (16) resulting in foetal hypo-perfusion associated with oxidative stress and low-grade inflammation (17–19). Studies have shown an imbalance of antioxidants and reactive oxygen species (ROS)-generating enzymes in pre-eclamptic placentas with endothelial dysfunction (20, 21). Furthermore, placental hypoxia which is common in PE has been shown to be associated with maternal endothelial dysfunction (22). These findings suggest PE to be associated with endothelial dysfunction, a precursor of CVDs. This has been evident in pre-eclamptic subjects compared to normotensive controls (23, 24). It is therefore possible that exposure of the unborn child to this pro-CVD environment may program the offspring for greater susceptibility to hypertension and CVDs (25, 26). Although Meeme et al. showed endothelial dysfunction to be associated with PE in rural South African women (27), it is still unknown whether children born to these pre-eclamptic mothers are at greater risk of CVDs. Hence, this protocol is for a study aiming to assess CVD risk profile of neonates and infants exposed to PE *in-utero* and correlate them with markers of maternal cardiovascular risk, oxidative stress and endothelial function. The study will:

1. Assess maternal cardiovascular risk profile during pregnancy
2. Assess cardiovascular risk profile of neonates at birth
3. Determine changes in cardiovascular risk profile from birth to 6 weeks after birth
4. Correlate the maternal and neonate CVD risk profile

## Methods and Analysis

### Ethical Approval and Informed Consent

Ethical approval was obtained from the Walter Sisulu University Human Research, Ethics and Biosafety Committee with approval number: 070/2021. Then the study was registered with the NIH ClinicalTrials.gov (Protocol https://ClinicalTrials.gov Identifier: NCT05091827; https://clinicaltrials.gov/ct2/show/NCT05091827). The purpose of the study will be explained thoroughly to potential participants attending the antenatal clinic in the Nelson Mandela Academic Hospital, Mthatha, Eastern Cape Province of South Africa. Pregnant women who meet the selection criteria and are willing to participate in the study will be required to sign informed consent forms for their participation and to allow their children to participate in the study from birth. Participation will be voluntary. This study will adhere to the standards of the South African National Data Protection Acts whereby the identity of the participants will be kept confidential. During data and sample collection, study ID will be assigned to participants to protect their identify and keep them confidential and anonymous. Participant's data will be stored in a secured database. After, commencement of study there will be no important changes in the methods.

### Selection Criteria

Pre-eclamptic or normotensive pregnant women with singleton, uncomplicated, between 20 and 26 weeks of pregnancy will be recruited for the study. Pregnant women with chronic hypertension, type 2 diabetes, gestational diabetes, renal and CVDs or any critical health condition will be excluded from the study. Furthermore, data will not be collected from eligible pregnant women who have performed physical exercise, smoked, or eaten for the past 6 h prior to data collection.

### Study Design and Procedure

This will be a prospective case-control study involving babies born to pre-eclamptic pregnant women and babies born to normotensive mothers recruited at the Nelson Mandela Academic Hospital, Mthatha, Eastern Cape Province of South Africa. Pregnant women who meet the selection criteria will be recruited between weeks 20–26 of gestation and their cardiovascular risk profile assessed at week 30 of gestation. The cardiovascular risk profile of their offspring will be assessed at birth and 6 weeks later.

Recruited pregnant women will be assessed at baseline (week 30 of gestation) for cardiometabolic risk profile including their PE status, anthropometry, blood pressure, oxidative stress, and vascular function. The socio-demographic, obstetric, and medical history will be collected using a structured interviewer administered questionnaire. Their blood pressure (systolic and diastolic blood pressures (BP) and heart rate, and anthropometric measures including weight and height will be measured. A vicorder (SMT Medical GmbH&Co., Wuerzburg, Germany) will be used to measure pulse wave velocity (PWV) to assess arterial stiffness and ankle-branchial index (ABI). The retinal microvasculature via retinal imaging will be assessed for vascular changes using a Optomed Aurora retinal camera (Optomed, Yrttipellotie 1, Finland). Gestational ultrasound will be performed to determine placental morphometry, architecture and vascularization. Fasting blood will be collected for the assessment of lipid profile, glycaemic, oxidative stress, reno-cardiovascular, and endothelial function markers.

At birth, the gestational age and mode of delivery of the offspring will be noted. The Appearance, Pulse, Grimace, Activity, and Respiration (APGAR) score (1–5 min) will be determined. The weight, height/length and blood pressure of the child will be measured, and PWV measured using a vicorder. Cord blood will be collected for determination of cardiometabolic risk markers including lipid profile, oxidative stress, reno-cardiovascular and endothelial function markers. Six weeks after birth, blood pressure (BP) and anthropometric measurements and PWV assessment will be done in children. Then, blood and urine samples will be collected for the assessment of endothelial function, lipid profile, reno-cardiovascular and oxidative stress markers.

**Figure 1:**
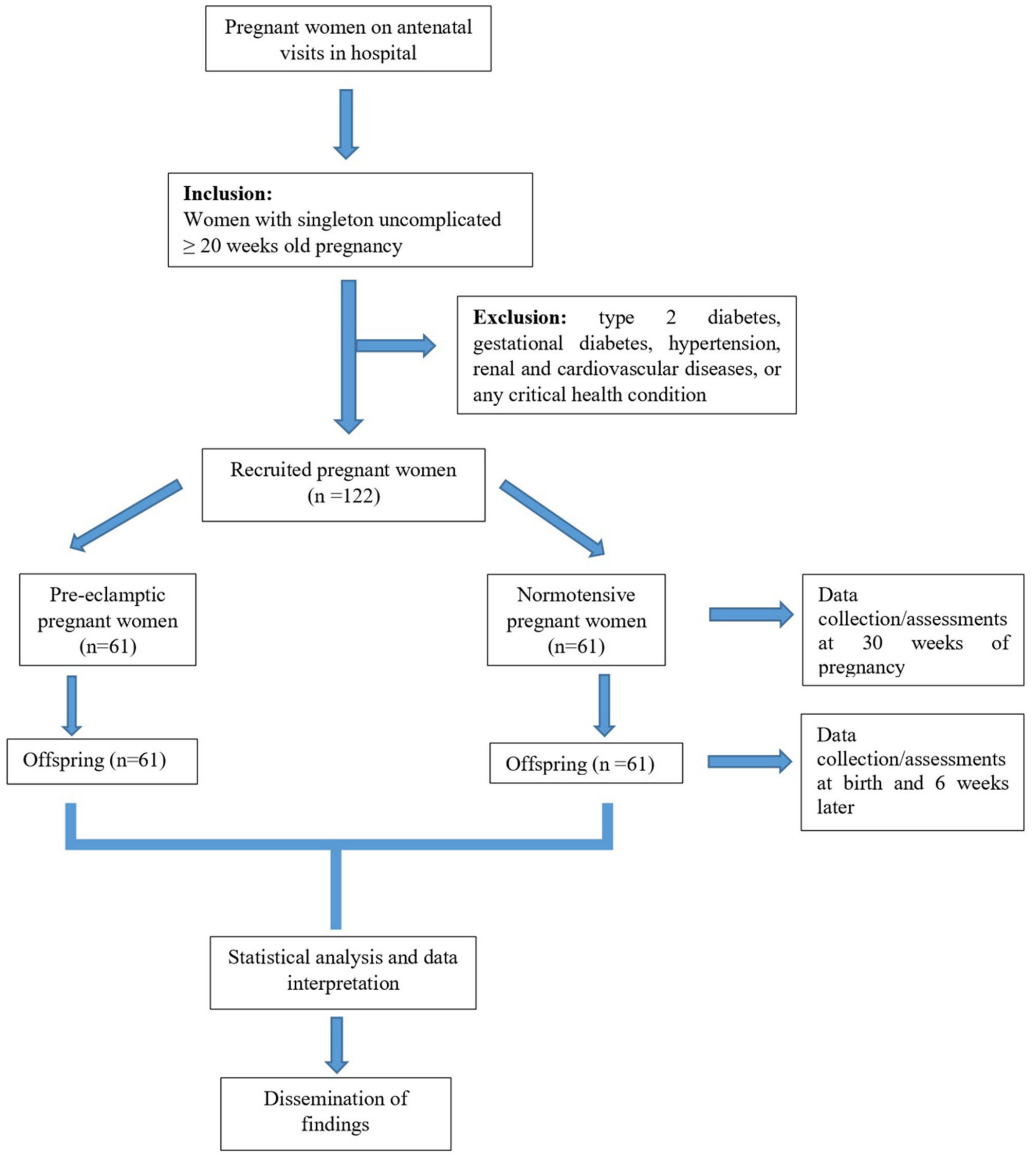
Study design flow chart.

**Figure 2:**
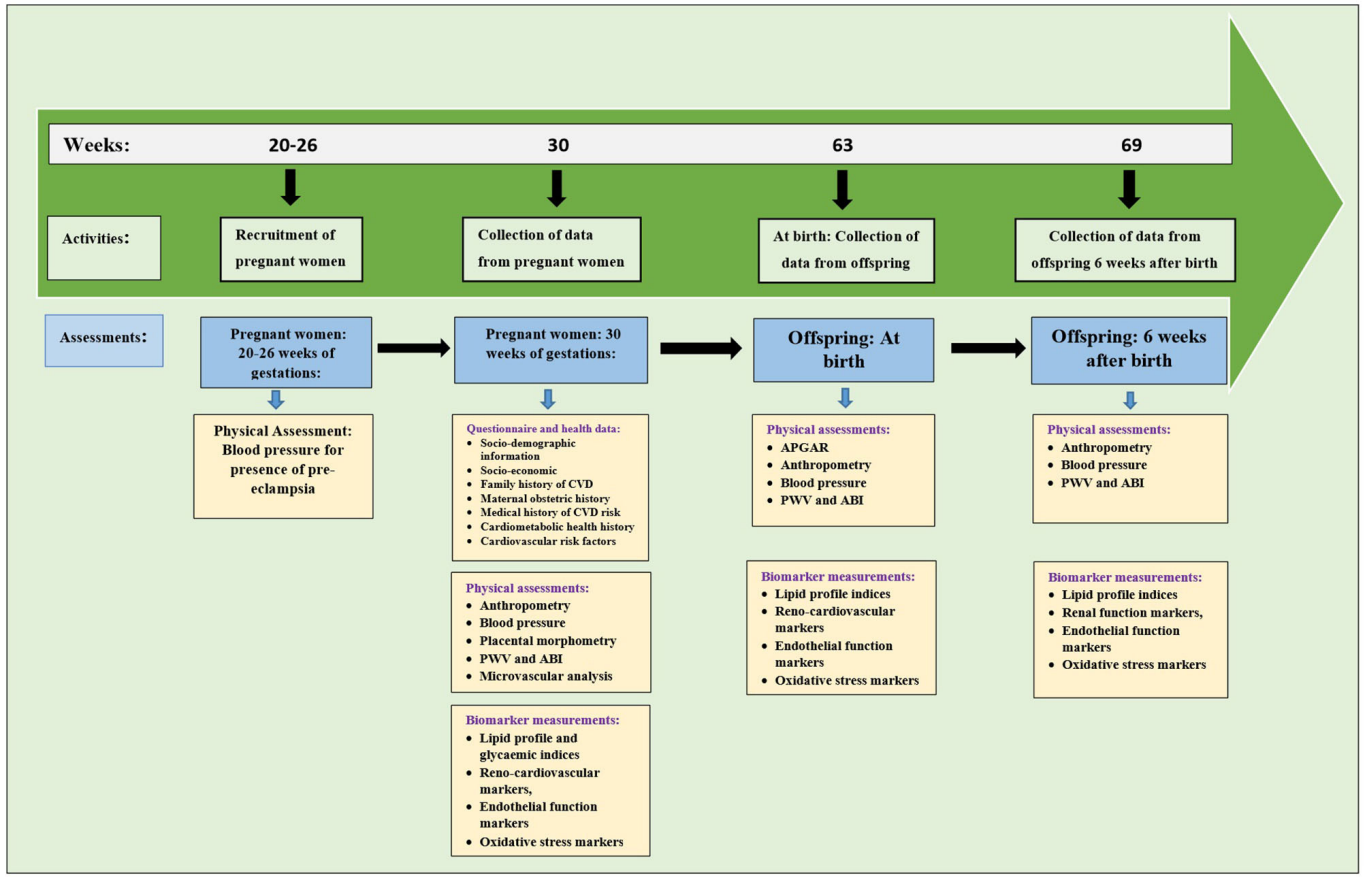
Timeline of the study along with physical and biomarkers assessments.

All pregnant women eligible for the study will be required to fast overnight and will be asked to restrain from smoking, drinking coffee, and physical exercise for 4–6 h prior to assessments. Recruitment of pregnant women at the hospital will commence in October 2021. The pregnant women will be followed-up for a period of 1 year. After delivery, follow-up of their offspring will commence in November 2022 and span through February 2023. Data collection and analysis will be completed by April 2023. The study design along with the various assessments and timeline are summarised in Figures 1, 2 respectively.

### Sample Size Calculation

The sample size was calculated with reference to the study by Amini et al. who showed BP differences between neonates from normotensive and pre-eclamptic mothers (28). The difference in systolic BP (SBP) was very high in pre-eclamptic mothers (68.2 ± 1.49 mmHg) compared to normotensive mothers (49.85 ± 5.49 mmHg). Because of the very high SBP variance and standard deviation in the pre-eclamptic group, we decided to use the diastolic blood pressure (DBP) values (normotensive: 30.17 ± 11.89 mmHg vs. pre-eclamptic: 42.11 ± 11.49 mmHg) for the sample size calculation. The software-R, version 4.1.0 (R Core Team, St. Louis, Missouri, USA) was used for the sample size calculation with parameters: alpha = 0.01, mean difference = 11.94 and standard deviation = 0.05. The calculated total sample size is 98 (49 cases and 49 controls). We assumed that not all participants would return for the follow-up and therefore adjusted our sample size by 20% to compensate for possible loses to follow-up. Thus, the total sample will be 98/(100–20/100) = 122 neonates. This implies that the study will commence with the recruitment of 122 pregnant women (61 pre-eclamptic women and 61 normotensive pregnant women) who meet the selection criteria.

### Data Collection

A standard interviewer administered structured questionnaire will be used to collect information on maternal health and cardiovascular risk factors at the beginning of the study (Supplementary file 1–Cardiovascular risk questionnaire). This information will include socio-demographics (sex, age, ethnicity, and location), socio-economic status (marital status, profession, income/revenue, and living standard), maternal obstetric history, family history, cardiometabolic health history, and cardiovascular risk factors (physical exercise, smoking, alcohol consumption, and feeding habit).

### Physical Assessments

Anthropometric measurements will be done in accordance with the international standards guidelines (29). A wall-mounted Harpenden stadiometer will be used to measure participants height to the nearest 0.1 cm while weight will be measured using a wireless weight scale (Tanita body composition scale). In pregnant women, BP including the SBP, DBP, and heart rate will be measured after a 10 min of rest in the seated position using the Omron M HEM-907XL Professional Blood Pressure Monitor (Omron Healthcare, Kyoto, Japan) described by Muntner et al. (30) in triplicates at 3 min intervals. The oscillometric technique (Dinamap 8100) will be used for BP measurement according to the standard protocol for assessment of BP measurement in newborns by Nwankwo et al. (31). An ultrasound device will be used to assess the morphometry, architecture and vasculature of the placenta. This will include assessing the uterine, umbilical and middle cerebral arteries and uterine artery mean pulsating index determined. Foetal cerebroplacental ratio and anthropometry will be calculated when possible.

### Assessment of Arterial Stiffness and Ankle-Branchial Index

A vicorder device (SMT Medical GmbH&Co., Wuerzburg, Germany) will be used for the assessment of pulse wave velocity (PWV), a measure for arterial stiffness and ankle-brachial index (ABI). Brachio-femoral PWV will be done as reported by Alwan et al. (32). Prior to PWV measurements, participants will be asked to lie in a supine position and rest for 10 min. Thereafter, a standard 10 cm pressure cuff will be placed on the upper right thigh as high as possible toward the crotch while another thin pressure cuff will be placed around the neck just on the carotid artery. The distance between the neck and thigh cuffs will be measured in cm using a measuring tape. Pressure lines: red pressure line to the neck cuff and blue pressure line to the thigh cuff, will be connected to the vicorder. Participants details including the BP, weight, height, and length between the carotid and femoral arteries will then be entered into the vicorder software and the test will be conducted. The PWV (m/s) will be calculated as a ratio of path length (between carotid and femoral arteries) and transit time. Prior to the measurement of ABI, a pressure cuff will be placed around the right arm of the participant and another pressure cuff around the ankle of the same side of the body. Participants details (BP, weight, and height) will be entered into the vicorder software while fixing the target SBP of 200 mmHg and the test conducted. The ABI will be calculated as the ratio of the SBP of the ankle and branchial regions.

### Microvascular Assessment by Retinal Imaging

The retinal microvasculature will be assessed for vascular changes via retinal imaging as previously reported (33). A digital retinal camera (Optomed, Yrttipellotie 1, Finland) will be used to capture the eye fundus for retinal vessel imaging. After capture of the right and left eye fundus, a trained grader, masked to participant's characteristics will perform vessel measurements on the optic disc–centred image. After measurements, a semi-automated IFLEXIS software (VITO, Belgium) will be used to analyse different retinal features including the microvascular state, the retinal vessel dimensions, and vascular tree (34, 35). This will be achieved by calculating the vessels widths and pattern features including the fractal analysis, lacunarity, and tortuosity. The average diameter of arterioles and venules of the eye will be summarised as central arteriolar equivalent (CRAE) and central retinal venular equivalent (CRVE), respectively (36).

### Biomarker Assessments

Blood and cord blood sample will be collected from pregnant women and early morning mid-stream urine samples will be collected from their offspring by the research nurse. These samples will be used to measure oxidative stress markers [lipid peroxidation, total antioxidant capacity, and 8-hydoxyl-2-deoxyguanine (8-OHdG)], lipid profile markers (total cholesterol, triglycerides, LDL-cholesterol, and HDL-cholesterol), insulin resistance markers [fasting glucose, insuli, and glycated haemoglobin (HbA1c)], endothelial function markers [endocan, nitric oxide (NO), and asymmetric dimethyl arginine (ADMA)] and renal function markers [glomerular filtration rate (GFR), creatinine and albumin] (37, 38). Fasting glucose and insulin will be used to calculate the homeostatic model assessment of insulin resistance (HOMA-IR) while urinary albumin and creatinine will be used to calculate the albumin to creatinine ratio (ACR) (37, 38).

### Statistical Analysis

STATA (Stata, College Station, Texas, USA) and IBM statistical package for social sciences (SPSS) Version 23.0 (IBM Corp). Armonk, NY, USA) will be used for data analyses. The Shapiro-Wilks test will be used to check for normality and data will be log-transformed and/or corrected for outliers when necessary. Descriptive statistics will be employed for data presentation as mean ± standard deviation (SD) or median ± interquartile range (IQR) for continuous variables and proportions (percentages) for categorical variables. Independent sample *t*-test will be used to compare mean differences between groups (pre-eclamptic and normotensive pregnant women and their offspring). Multivariate analysis using multiple analysis of variance (MANOVA) will be used to compare continuous variables for two or more independent variables where necessary. Chi-square test of association and Pearson correlation will be used to assess the relationship between cardiometabolic risk factors and PE at baseline and follow-up for both mother and child. A difference will be considered significant at α = 0.05.

## Discussion

This study aims to investigate the relationship between PE and cardiovascular risk in children born to pre-eclamptic mothers in a rural South African population of African descents. It has been suggested that the foeto-placental vascular endothelial dysfunction may cause epigenetic alteration in the intrauterine environment of the foetus (39). Therefore, the maternal gestational environment may be programmed to have a significant impact on foetal and offspring health later in life (9). In fact, some studies have undertaken to investigate whether children born to pre-eclamptic mothers may predispose them to cardiovascular risk factors. Miller et al. demonstrated as early as 1983 that full-term neonates born to pre-eclamptic mothers have transient hypertension during the first hours of life (40). A meta-analysis study with over 45,000 individuals reported a 1.35 mmHg and 2.39 mmHg higher DBP and SBP in children and young adults born to pre-eclamptic pregnancies (41). The timing of the onset of PE has been reported to be a determinant of hypertension in offspring later on in life as offspring born to mothers with early PE onset were found to have increased central and peripheral SBP (6 mmHg) (42). Also, offspring of pre-eclamptic pregnancies showed a distinct vascular phenotype which could possibly mediate increased risk of hypertension in future (13). More so, impaired flow mediated endothelial responses as well as increased carotid intima-media thickness which suggest endothelial dysfunction was observed in preterm-born young adults exposed to a hypertensive pregnancy (12). A Spanish study on *in-utero* growth restricted children showed that children born to pre-eclamptic parents had higher BP, cardiac remodeling, and evidence of endothelial dysfunction at 6 months of age. Follow-up studies on school age children showed that they had higher SBP and DBP, smaller hearts, increased heart rate and features of cardiac diastolic dysfunction (43). A Norway study of 15,778 participants of which 343 were born to pre-eclamptic mothers showed increased BP, body mass index, wider waist circumference, higher non–HDL-cholesterol and triglycerides suggesting increased risk of CVDs (44). All these studies have shown that children born to pre-eclamptic mothers are prone to obesity, hypertension, diabetes and CVDs in their adult life (45, 46). However, all these findings were established in western populations with little or no reports from children of African origin. To our knowledge there is yet to be such a study in an African population. Conversely, it remains unclear whether the high prevalence of cardiovascular risk factors (obesity, hypertension etc) observed in South African children of African ancestry (47, 48) may have originated from pre-eclamptic mothers. The assessment of cardiovascular risk in pre-eclamptic pregnant women and the follow-up of their offspring after birth for possible cardiovascular risk may address this concern.

The findings from this study will provide information on the cardiovascular risk profile of offspring of pre-eclamptic mothers of African ancestry living on the African continent. It will also provide an opportunity to understand the relationship between the maternal environment and foetus, and how this may influence the risk for CVDs. A knowledge of the risk profile of the offspring of pre-eclamptic mothers may be used to develop plans/programs for CVD prevention in the offspring of women with PE. Also, findings from this study may help to develop policy around maternal and child health to ensure CVD risk prevention in both pregnant women with PE and children born to them. For the purpose of primary prevention of CVDs in children and later in life, it is important to identify predisposing risk factors in pregnant women so that intervention strategies for preventing intrauterine programming as a result of PE may be developed. This may give insight on lifestyle modifications that may be useful for the mother and child both during pregnancy and after birth. To our knowledge, this will be the first longitudinal study to assess the risk factors for CVD *in-utero* and at two time points after birth in a population of pre-eclamptic women of African ancestry. This will be the first study to assess vascular function and its markers in children exposed to the PE environment *in-utero* in an African community and done in children of African ancestry.

Despite the strengths of this study, we will also want to acknowledge some limitations of the study. This study will be conducted in Mthatha in the Eastern Cape Province of South Africa, a region highly dominated by African Ancestry population. Other ethnic populations within South Africa will not be considered. However, the ethnicity of the children born of these mothers may not be restricted because of the background of their fathers. The prevalence of PE in South Africa is very low, and therefore, recruitment of patients will be challenging. To curb this challenge, we will work with medical personnel at the recruiting hospital to alert us on eligible patients during antenatal visits. The study intends to follow-up offspring after birth for a period of 6 weeks which may not be sufficient to assess the progression of cardiovascular risk in children. However, this limitation in the short follow-up period was with respect to the available funding for the project. Ultrasound-based flow-mediated dilatation (FMD) measurements of the brachial artery which is known to be the goal standard for the assessment of endothelial function (49, 50) will not be used for this study as this equipment requires participants to be stable during assessment, but such stable state may not be achieved during pregnancy especially when pregnant women are in their 3rd trimester experiencing labour (uterus contractions). As such, endothelial function assessments will be limited to biomarkers of endothelial function. As reported by the developmental origin of health and diseases (DOHaD) certain drugs have been reported to affect the in-uterine environment and/or the foeto-placental endothelium which may predispose offspring to cardiovascular risk factors (51). This study will not be able to control the effects of drugs that can affect the *in-utero* environment and foeto-placental endothelium. To address this concern pregnant women will report all drugs that they had taken during pregnancy and their duration. Our statistician will be able to identify such drugs with long duration that could affect the study outcome and will be considered as confounding variable during statistical analysis.

In all, this study will seek to address the possible relationship between PE and cardiovascular risk in children born to pre-eclamptic mothers in South Africans of African ancestry. This study will be of importance to the South African population as there is evidence of rising prevalence of cardiovascular risk factors such as obesity, hypertension, diabetes and metabolic syndrome observed in South African children without a clear understanding of the underlining cause. The findings of this study may contribute to the development of policies that can help in the prevention of CVD risk in pre-eclamptic mother and their children. It may also help to develop intervention strategies for the prevention of intrauterine programming resulting from PE in pregnancy.

## Ethics Statement

This study involving human participants was reviewed and approved by Walter Sisulu University Human Research, Ethics and Biosafety Committee with approval number: 070/2021. The patients/participants will provide their written informed consent to participate in this study.

## Author Contributions

BN-C and GE conceptualised the research project and wrote the manuscript. BN-C, GE, CB, NG, KK-H, and AK design the study and developed the questionnaire. BN-C, GE, CB, AK, and NG wrote the original grant proposal. CB, KK-H, AK, and NG reviewed and edited the manuscript. All authors approved the final manuscript.

## Funding

This study was supported by the South African Medical Research Council (SAMRC) with funds received from South African Department of Science and Innovation awarded to BN-C.

## Conflict of Interest

The authors declare that the research was conducted in the absence of any commercial or financial relationships that could be construed as a potential conflict of interest.

## Publisher's Note

All claims expressed in this article are solely those of the authors and do not necessarily represent those of their affiliated organizations, or those of the publisher, the editors and the reviewers. Any product that may be evaluated in this article, or claim that may be made by its manufacturer, is not guaranteed or endorsed by the publisher.
