# Supplementary material for: Assessment of the Cardiovascular Risk Profile of Infants Exposed to Pre-eclampsia in-utero: A Prospective Case-Control Study in South African Children of African Ancestry
Source: Front Cardiovasc Med. 2021 Nov 23;8:773841. doi: 10.3389/fcvm.2021.773841 (PMC8650009; doi:10.3389/fcvm.2021.773841)
Supplement: Supplementary file 1 [file Data_Sheet_1.pdf]

ASSESSMENT OF THE CARDIOVASCULAR RISK PROFILE OF INFANTS EXPOSED TO PRE-ECLAMPSIA *IN-UTERO*: A PROSPECTIVE CASE-CONTROL STUDY IN SOUTH AFRICAN CHILDREN OF AFRICAN ANCESTRY

MATERNAL INFORMATION

DEMOGRAPHICS

Patient ID:	___________________________________
Firstname	___________________________________
Surname	___________________________________
House/Flat Number	___________________________________
Dwelling type	o House<br>o Flat or Complex<br>o Informal housing
Building/Complex Name	___________________________________
Street name	___________________________________
Suburb	___________________________________
For how many years have you lived at current address?	___________________________________<br>((months as decimal e.g. 6 months = 0.6))
Telephone number	___________________________________
Age	___________________________________

PREGNANCY

Are you pregnant	o Yes<br>o No
If pregnant, how old is your pregnancy	___________________________________<br>((weeks))
If pregnant what trimester is your pregnancy	1st (1-11 weeks/1-3 months)<br>2nd (12-23 weeks/3-6 months)<br>3rd (above 24 weeks/ above 6 months)
Is this your first pregnancy?	o Yes<br>o No
If No, state the number of times you have been pregnant	[___]
State your age at first pregnancy	[___]year
Have you ever had stillbirth?	o Yes<br>o No
State the number of Children you have?	[___]
What is the spacing between your children?	[___]

State the drugs taken during pregnancy and the duration in weeks	Drug 1:_______________. Duration (Wks):_______.<br>Drug 2:_______________. Duration (Wks):_______.<br>Drug 3:_______________. Duration (Wks):_______.<br>Drug 4:_______________. Duration (Wks):_______.

PRE-ECLAMPSIA STATUS AND RISK FACTORS

Do you have pre-eclampsia?	○ Yes<br>○ No
If this is not your first pregnancy, have you ever had pre-eclampsia?	○ Yes<br>○ No
Do you have chronic hypertension?	○ Yes<br>○ No
Has any person in your family had pre-eclampsia? That is your mother or sisters?	○ Yes<br>○ No
Have you ever been pregnant through assisted reproductive technology?	○ Yes<br>○ No
Have you ever had chronic kidney disease?	○ Yes<br>○ No

HIV

Are you HIV positive?	○ Yes<br>○ No
If positive, are you on ART?	○ Yes<br>○ No
Which line of ART are you on?	1st 2nd
What is the name of the ART?	____________________________________

CARDIOVASCULAR RISK

Do you have high blood pressure (hypertension)?	○ Yes<br>○ No<br>○ Unknown
If yes, what year were you first diagnosed?	____________________________________
Do you have a heart disease or stoke?	○ Yes<br>○ No<br>○ Unknown
If yes, what year were you diagnosed?	____________________________________

DIABETES

Do you have Diabetes	○ Yes<br>○ No
What type of Diabetes do you have?	○ Type I Diabetes (also known as Juvenile Onset or Insulin Dependent Diabetes)<br>○ Type II Diabetes (also known as Non-insulin Dependent Diabetes)<br>○ Gestational diabetes<br>○ Don't know

FAMILY HISTORY

STROKE AND HEART DISEASE

	Don't know	No	Yes, under the age of 60	Yes, over the age of 60	Yes, but I don't know the age
Has your mother had any heart disease?					
Has your father had any heart disease?					
Has your mother ever had a stroke?					
Has your father ever had a stroke?					

DIABETES

	Don't know	No	Yes, Type I	Yes, Type II	Yes, but I don't know type
Has your mother had any Diabetes?					
Has your father had any Diabetes?					

HIGH BLOOD PRESSURE

	No	Yes	Don't know
Has your mother had high blood pressure?			
Has your father had high blood pressure?			

LIFESTYLE

Are you a smoker	○ Yes currently<br>○ In the past<br>○ Never smoked
What type of cigarette do/did you smoke?	○ Snuf<br>○ Tobacco<br>○ Dagga
On average, how many cigarettes do you smoke on the days that you smoke?	○ More than 20 daily<br>○ Less than 20 daily
If you have stopped, how long has it been since you last smoked (months)?	___________________________________

ALCOHOL

Have you consumed an alcoholic drink within the past 12 months?	○ Yes<br>○ No
How often do you typically drink ?	○ Daily<br>○ 8 or more days a month<br>○ Less than 8 days a month
At what age did you start drinking regularly (at least once a week)?	___________________________________<br>((answer in years))

ANTHROPOMETRY

Total body mass	___________________________________(kg)
Height	___________________________________(cm)
Body Mass Index (BMI)	___________________________________
Hip circumference	___________________________________(cm)
Waist circumference	___________________________________(cm)
Waist to hip ratio	___________________________________

BLOOD PRESSURE

Systolic	___________________________________
Diastolic	___________________________________
Arm	○ Left<br>○ Right
Heart Rate	___________________________________(bpm)

Systolic (2nd reading)	____________________________________
Diastolic (2nd reading)	____________________________________
Arm (2nd reading)	○ Left<br>○ Right
Heart rate (2nd reading)	____________________________________(bpm)
Systolic (3rd reading)	____________________________________
Diastolic (3rd reading)	____________________________________
Arm (3rd reading)	○ Left<br>○ Right
Heart Rate (3rd reading)	____________________________________(bpm)
Mean Blood Pressure	____________________________________
Mean Heart Rate	____________________________________

PLACENTAL MORPHOMETRY, ARCHITECTURE AND VASCULARIZATION

Uterine arteries,	
Umbilical artery	
Middle cerebral artery and	
Uterine artery mean pulsating index	
Foetal cerebroplacental ratio	

NEONATES INFORMATION

DEMOGRAPHIC

Sex	____________________________________
Date of birth	____________________________________
Time of birth	____________________________________
	____________________________________
Mode of delivery	____________________________________
APGAR Score	○ 1<br>○ 2<br>○ 3<br>○ 4<br>○ 5

ANTHROPOMETRY

Weight	___________________________________(kg)
Height	___________________________________(cm)
Weight/height ratio	___________________________________
Head circumference	___________________________________(cm)
Chest circumference	___________________________________(cm)
Abdominal circumference	___________________________________(cm)
Mid upper arm circumference	___________________________________(cm)
Fore arm circumference	___________________________________(cm)

BLOOD PRESSURE

Systolic	___________________________________
Diastolic	___________________________________
Arm	○ Left<br>○ Right
Heart Rate	___________________________________(bpm)
Systolic (2nd reading)	___________________________________
Diastolic (2nd reading)	___________________________________
Arm (2nd reading)	○ Left<br>○ Right
Heart rate (2nd reading)	___________________________________(bpm)
Systolic (3rd reading)	___________________________________
Diastolic (3rd reading)	___________________________________
Arm (3rd reading)	○ Left<br>○ Right
Heart Rate (3rd reading)	___________________________________(bpm)
Mean Blood Pressure	___________________________________
Mean Heart Rate	___________________________________
